# Sesquiterpene Lactone Derivatives of *Ambrosia artemisiifolia* Act as Agonists of the Transient Receptor Potential Ankyrin 1 Ion Channel

**DOI:** 10.3390/ph19091404

**Published:** 2026-09-06

**Authors:** Balázs Zoltán Zsidó, Csaba Hetényi, Balázs Kovács, Dezső Csupor, Tivadar Kiss, Boglárka Csupor-Löffler, Zsuzsanna Helyes, Éva Szőke

**Affiliations:** 1 Department of Pharmacology and Pharmacotherapy, Medical School, University of Pécs, Szigeti u. 12, H-7624 Pécs, Hungary; hetenyi.csaba@pte.hu (C.H.); helyes.zsuzsanna@pte.hu (Z.H.); eva.szoke@aok.pte.hu (É.S.); 2 National Laboratory for Drug Research and Development, Magyar Tudósok krt. 2, H-1117 Budapest, Hungary; 3 Faculty of Pharmacy, Institute of Pharmacognosy, University of Szeged, Eötvös u. 6, H-6720 Szeged, Hungary; kovex92@gmail.com (B.K.); csupor.dezso@gmail.com (D.C.); kiss.tivadar@szte.hu (T.K.); 4 Faculty of Pharmacy, Institute of Clinical Pharmacy, University of Szeged, Szikra u. 8, H-6720 Szeged, Hungary; 5 Institute of Translational Medicine, Medical School, University of Pécs, Szigeti u. 12, H-7624 Pécs, Hungary; 6 HUN-REN SZTE Biologically Active Natural Products Research Group, Eötvös u. 6, H-6720 Szeged, Hungary; 7 Department of Pharmaceutics, Faculty of Pharmacy, University of Pécs, Rókus u. 2, H-7624 Pécs, Hungary; csupor-loffler.boglarka@pte.hu; 8 HUN-REN PTE Chronic Pain Research Group, Szigeti út 12, H-7624 Pécs, Hungary; 9 PharmInVivo Ltd., Szondi u. 10, H-7629 Pécs, Hungary

**Keywords:** TRPA1, sesquiterpene, ragweed, covalent, agonist

## Abstract

**Background:** Transient receptor potential ankyrin 1 (TRPA1) and vanilloid 1 (TRPV1) are key nociceptive ion channels involved in chemosensation, pain signaling, and neurogenic inflammation. Several sesquiterpene lactones target TRPA1, but the activity profile of these secondary metabolites of the common ragweed (*Ambrosia artemisiifolia*) remains uncharacterized. Purpose: The purpose of this study was to explore the activity of five ragweed sesquiterpene lactones on TRPA1 and TRPV1, respectively. **Methods:** We investigated the effects of five ragweed-derived sesquiterpene lactones—acetoxydihydrodamsin, costunolide, isoalantolactone, peruvin, and psilostachyin—on TRPA1 and TRPV1 using ^45^Ca^2+^ uptake in receptor-overexpressing CHO cells and covalent docking to human TRPA1. **Results:** Costunolide, isoalantolactone and acetoxydihydrodamsin induced TRPA1 activation (counts per minute of ^45^Ca^2+^ uptake at 40 µM concentration: 5070 ± 346.5, 10,197 ± 1237, 3704 ± 1634, respectively) but not TRPV1 activation, as demonstrated by Ca^2+^ influx. Acetoxydihydrodamsin induced concentration-dependent TRPA1 activation that was significantly inhibited by 10 µM of the selective antagonist HC-030031. Docking studies demonstrated covalent interactions of costunolide, isoalantolactone, and acetoxydihydrodamsin (−47.3, −51.5, and −45.0 kcal/mol FITTED score) with the electrophile-sensitive binding region of TRPA1. **Conclusions:** Ragweed sesquiterpene lactones act as TRPA1 agonists without detectable TRPV1 activation. This study provides the first data identifying acetoxydihydrodamsin as a TRPA1 agonist, expanding the pharmacological map of ragweed sesquiterpene lactones. These results suggest that these metabolites may contribute to both the irritant properties and the pharmacological potentials of *A. artemisiifolia*.

## 1. Introduction

Transient receptor potential ankyrin 1 (TRPA1) and transient receptor potential vanilloid 1 (TRPV1) are polymodal, Ca^2+^-permeable, non-selective cation channels that play central roles in chemosensation, nociception, and neurogenic inflammation [1]. TRPA1 is widely expressed in primary sensory neurons, including dorsal root, trigeminal, and nodose ganglia, and is activated by a broad spectrum of exogenous and endogenous irritants. Electrophilic ligands may activate the channel through covalent binding to cysteine residues [2,3,4,5,6], whereas non-electrophilic agonists act through non-covalent interactions [7]. TRPA1 is activated by noxious cold (T < 17 °C) and numerous endogenous or exogenous compounds, such as allyl isothiocyanate (AITC in mustard oil), menthol, and formaldehyde [8,9,10,11,12,13,14,15]. TRPV1 is activated by capsaicin, noxious heat, protons, and several endogenous inflammatory mediators. They are commonly co-localized in sensory neurons and play key roles in pain signaling and inflammation [8,16,17,18,19]. Pro-inflammatory neuropeptides (calcitonin gene-related peptide: CGRP, Substance P) are released in response to their activation, which induce vasodilation, plasma protein extravasation and immune cell activation, collectively called neurogenic inflammation [8,20,21,22,23]. TRPA1 contributes to allergen-induced neurogenic airway inflammation, and the severity of allergic response in urban settings may be exacerbated by chronic exposure to TRPA1-activating air pollutants such as formaldehyde and ozone [24]. TRPV1 also contributes to respiratory neurogenic inflammation by promoting neuropeptide release from peptidergic sensory fibers [25]. It was shown in an airway inflammatory animal model that the expression levels of TRPA1 and inflammatory cytokine IL-1ß are increased [26]. In turn, the inhibition of TRPA1 or TRPV1 reduces the activity of the NOD-like receptor thermal protein domain-associated protein 3/cysteinyl aspartate specific proteinase (NLRP3/caspase-1) inflammatory pathway and consequential IL-1ß production [27]. Another role of TRPA1 in inflammation is through the suppression of the tumorigenicity two receptor (ST2) pathway [28]. Macrophages produce IL-33 in ankle inflammation, which acts on its receptor ST2, promoting neutrophil influx and neutrophil-dependent reactive oxygen species (ROS) production, activating TRPA1 in dorsal root ganglion (DRG) neurons. The DRG neurons innervate the inflamed ankle and produce nociception exacerbated by ROS [28]. 

Natural products have emerged as valuable tools for investigating TRP channel pharmacology. A wide variety of plant-derived metabolites, including irritants and pungent compounds, activate TRPA1, and this channel is now recognized as a major molecular target for chemically diverse defensive metabolites produced across the plant kingdom. Within this context, sesquiterpenes and sesquiterpene lactones are especially intriguing. Deterrent sesquiterpenes such as polygodial and isovelleral activate TRPA1 and account for noxious, pungent, and inflammatory responses in vivo, establishing this channel as a key sensor for reactive terpenoid natural products [29]. Electrophilic natural products, including Michael acceptors, are of particular interest because their thiol reactivity can underlie biologically relevant yet mechanistically diverse interactions with protein nucleophiles [30], such as the thiol (-SH) group of a cysteine amino acid. The active site cysteines (e.g., C621) of TRPA1 have a reactive thiol group that can be attacked by electrophilic agonists to form a covalent bond with the receptor. Higher thiol reactivity in an agonist means that it is more likely to interact with the active site cysteine of TRPA1.

Sesquiterpene lactones harboring an alpha-methylene-gamma-lactone moiety are potent Michael acceptors and effective TRPA1 agonists. Consistent with this concept, parthenolide, a major secondary metabolite of Tanacetum parthenium, was shown to selectively activate TRPA1 as a partial agonist, thereby inducing channel desensitization and the non-selective de-functionalization of peptidergic sensory neurons [31]. Isopetasin, a sesquiterpene from Petasites hybridus, induced neuronal desensitization via TRPA1 activation [32]. In *Waldheimia glabra,* (+)-ludartin was identified as a natural TRPA1 agonist [33]. The activity of several sesquiterpene lactones from the well-known allergen *Ambrosia artemisiifolia* L. (Asteraceae, common ragweed) has been studied in vitro. The TRPA1 agonist activity of isabelin outperformed that of costunolide and isoalantolactone, and the potency of these compounds correlated with their thiol-reactive Michael acceptor character [34]. Neoambrosin acted as a partial agonist of TRPA1 channels; however, damsin showed no activity [35]. These observations suggest that sesquiterpene lactones are not only abundant and chemically diverse plant metabolites but also biologically relevant ligands for chemosensory ion channels. 

**Figure 1:**
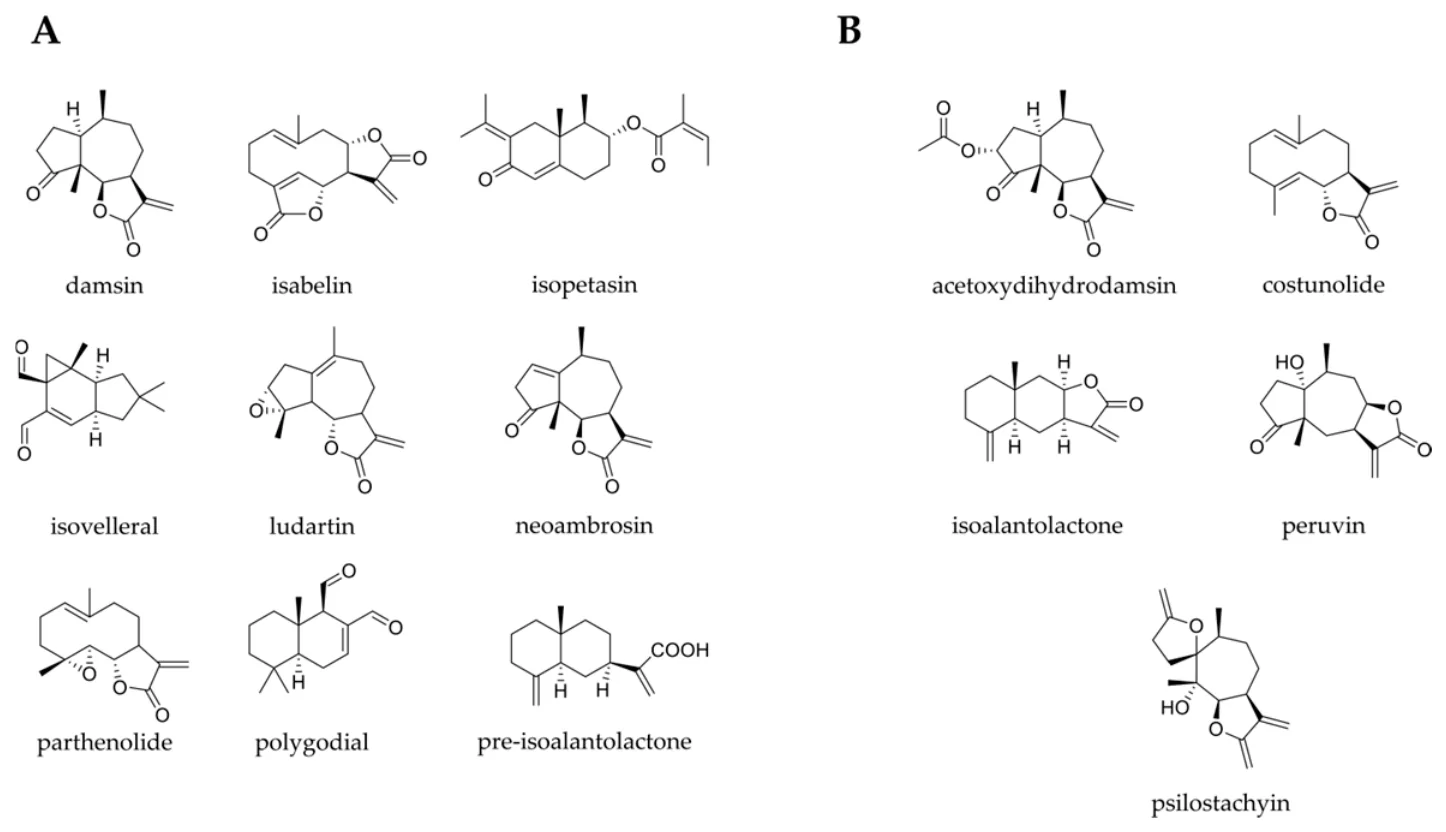
Chemical structures of sesquiterpenoids relevant to present study. (**A**) Representative naturally occurring sesquiterpenoids previously investigated as TRPA1 modulators: damsin, isabelin, isopetasin, isovelleral, (+)-ludartin, neoambrosin, parthenolide, polygodial, and pre-isoalantolactone. (**B**) Sesquiterpene lactones of *Ambrosia artemisiifolia* investigated in present study: acetoxydihydrodamsin, costunolide, isoalantolactone, peruvin, and psilostachyin.

The chemical structures of the representative sesquiterpenoids discussed above, together with those of the five *A. artemisiifolia* sesquiterpene lactones investigated in the present study, are shown in Figure 1 to facilitate a comparison of their structural features and electrophilic functionalities.

Recent data indicate that structurally similar sesquiterpenes may engage TRP channels through distinct mechanisms. For example, isoalantolactone acts as a classical thiol-reactive TRPA1 agonist, whereas its closely related acidic precursor, pre-isoalantolactone, can still activate TRPA1 despite lacking detectable thiol reactivity, suggesting a different, likely non-covalent mode of engagement [34]. Nevertheless, the TRP channel pharmacology of the characteristic sesquiterpene lactones of A. artemisiifolia remains only partially explored. Although TRPA1 activity has been demonstrated for selected ragweed sesquiterpenoids, including costunolide and isoalantolactone [34], other prominent constituents, such as acetoxydihydrodamsin, peruvin, and psilostachyin, have not been systematically characterized in this respect. Moreover, their activity at TRPV1, another major nociceptive TRP channel, is largely unknown. This represents a relevant knowledge gap because even closely related sesquiterpene lactones may differ substantially in electrophilic reactivity and, consequently, in their ability and mechanism to activate TRPA1.

Sesquiterpenes of the genus *Ambrosia* have been reported to exhibit a broad range of biological activities, with antiproliferative, cytotoxic, and antiprotozoal effects being among the most extensively investigated [36]. Earlier, we isolated acetoxydihydrodamsin, 1′-noraltamisin, peruvin, psilostachyin, psilostachyin B, and psilostachyin C from *A. artemisiifolia* and demonstrated the cytotoxic and antiproliferative activities of several of these compounds on tumor cell lines [37]. Acetoxydihydrodamsin showed particularly pronounced cytotoxic and antiproliferative effects, whereas psilostachyin also displayed marked antiproliferative activity [37]. Psilostachyin and peruvin have additionally been reported to possess antiprotozoal activity against *Trypanosoma*, *Leishmania*, and *Plasmodium* species [36]. The anti-inflammatory and antiproliferative effects of costunolide and isoalantolactone have been extensively reported [38].

Earlier, we isolated the sesquiterpene lactones acetoxydihydrodamsin, 1′-noraltamisin, peruvin, psilostachyin, psilostachyin B, and psilostachyin C and demonstrated their cytotoxic and antiproliferative activities on tumor cell lines [37]. In a subsequent quantitative phytochemical study, we identified acetoxydihydrodamsin, costunolide, isoalantolactone, peruvin, and psilostachyin as characteristic sesquiterpene lactones of A. artemisiifolia and quantified their levels during the whole vegetational period [39]. These five compounds were therefore selected for the present study to represent the characteristic sesquiterpene lactone profile of the plant. The inclusion of costunolide and isoalantolactone, whose TRPA1 agonist activity had previously been demonstrated [34], also provided reference compounds against which the less characterized ragweed constituents could be compared. Here, we report the effects of acetoxydihydrodamsin, costunolide, isoalantolactone, peruvin, and psilostachyin on TRPA1 and TRPV1. By comparing structurally related ragweed sesquiterpene lactones with different electrophilic properties, we aimed to expand the pharmacological map of *A. artemisiifolia* constituents at these two major nociceptive targets. Furthermore, we aim to explore the mechanism of TRPA1 activation by various sesquiterpene covalent warheads.

## 2. Results

### 2.1. TRPA1 but Not TRPV1 Activation Induces Radioactive ^45^Ca^2+^ Uptake in Receptor-Overexpressing CHO Cells 

**Figure 2:**
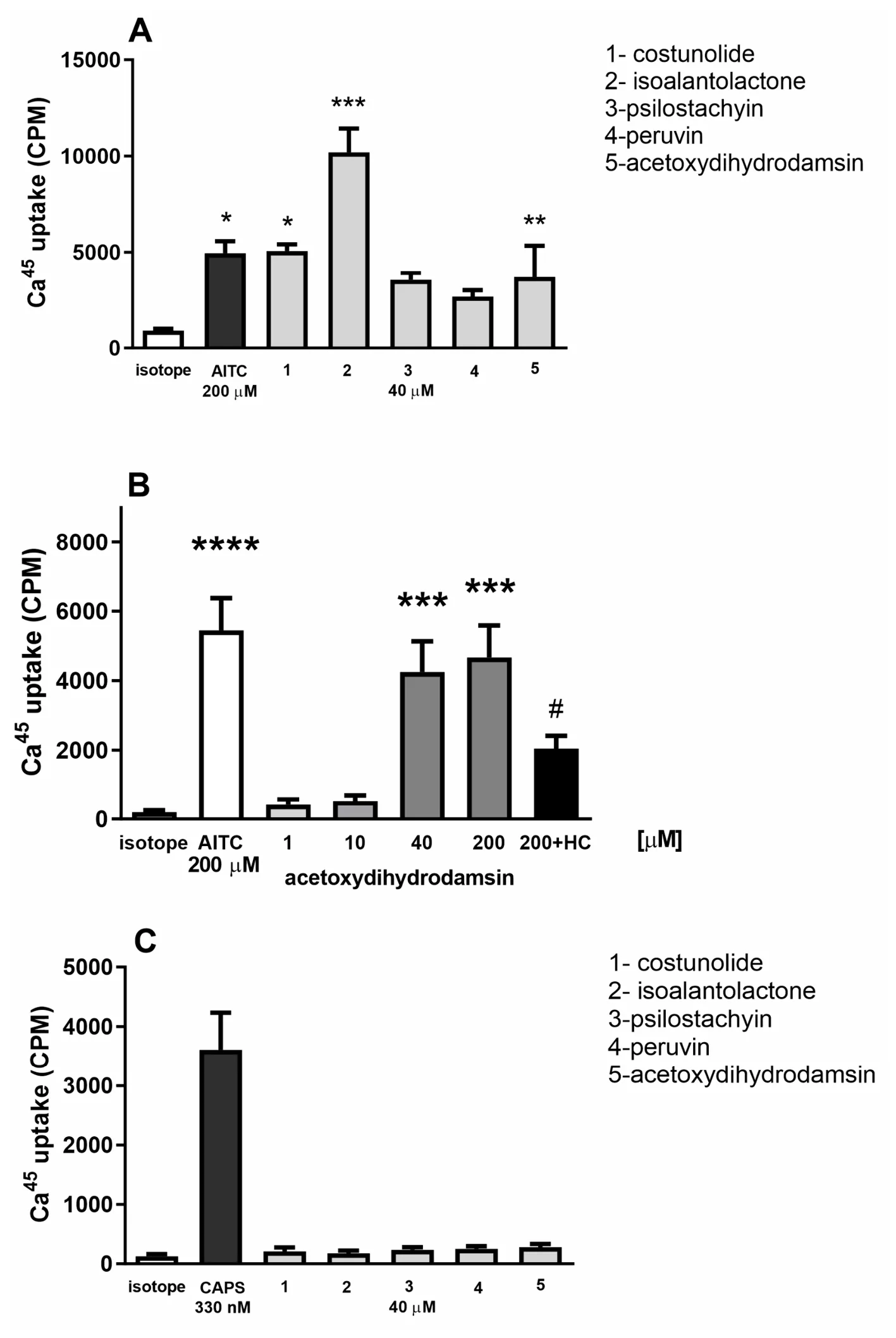
Effect of costunolide, isoalantolactone, psilostachyin, peruvin and acetoxydihydrodamsin on ^45^Ca^2+^ uptake of CHO cells expressing cloned TRPA1 (**A**,**B**) and TRPV1 (**C**) receptors. ^45^Ca^2+^ accumulations are presented as counts per minute (CPM). Each column represents mean ± SEM from three independent experiments, each performed in triplicate (total *n* = 9 measurements per treatment group), * *p* < 0.1, ** *p* < 0.01, *** *p* < 0.001, **** *p* < 0.0001 (vs. control, one-way ANOVA, Dunnett’s post hoc test), # *p* < 0.05 (vs. 200 µM acetoxydihydrodamsin).

The positive control TRPA1 agonist AITC (200 µM) resulted in 4950 ± 635.8 CPM ^45^Ca^2+^ influx into TRPA1-expressing cells. In the first series of experiments, 40 µM costunolide, isoalantolactone, and acetoxydihydrodamsin, but not psilostachyin and peruvin, caused significant ^45^Ca^2+^ influx (CPM values: 5070 ± 346.5, 10,197 ± 1237, 3704 ± 1634, respectively) (Figure 2A). Then, acetoxydihydrodamsin-induced ^45^Ca^2+^ uptake was investigated in a concentration-dependent manner (1, 10, 40, and 200 µM). Acetoxydihydrodamsin induced significant ^45^Ca^2+^ uptake at 40 and 200 µM, which was significantly decreased by the TRPA1 antagonist HC-030031 (10 µM) (Figure 2B). The reference TRPV1 agonist capsaicin (330 nM) resulted in 3610 ± 625.8 CPM ^45^Ca^2+^ influx into TRPV1-expressing cells, but none of the tested compounds caused TRPV1 activation (Figure 2C). The retention of ^45^Ca^2+^ was negligible in Hank’s solution plus isotope controls.

### 2.2. Isoalantolactone and Acetoxydihydrodamsin Induce TRPA1-Mediated Ca^2+^ Influx in Cultured TRG Neurons 

**Figure 3:**
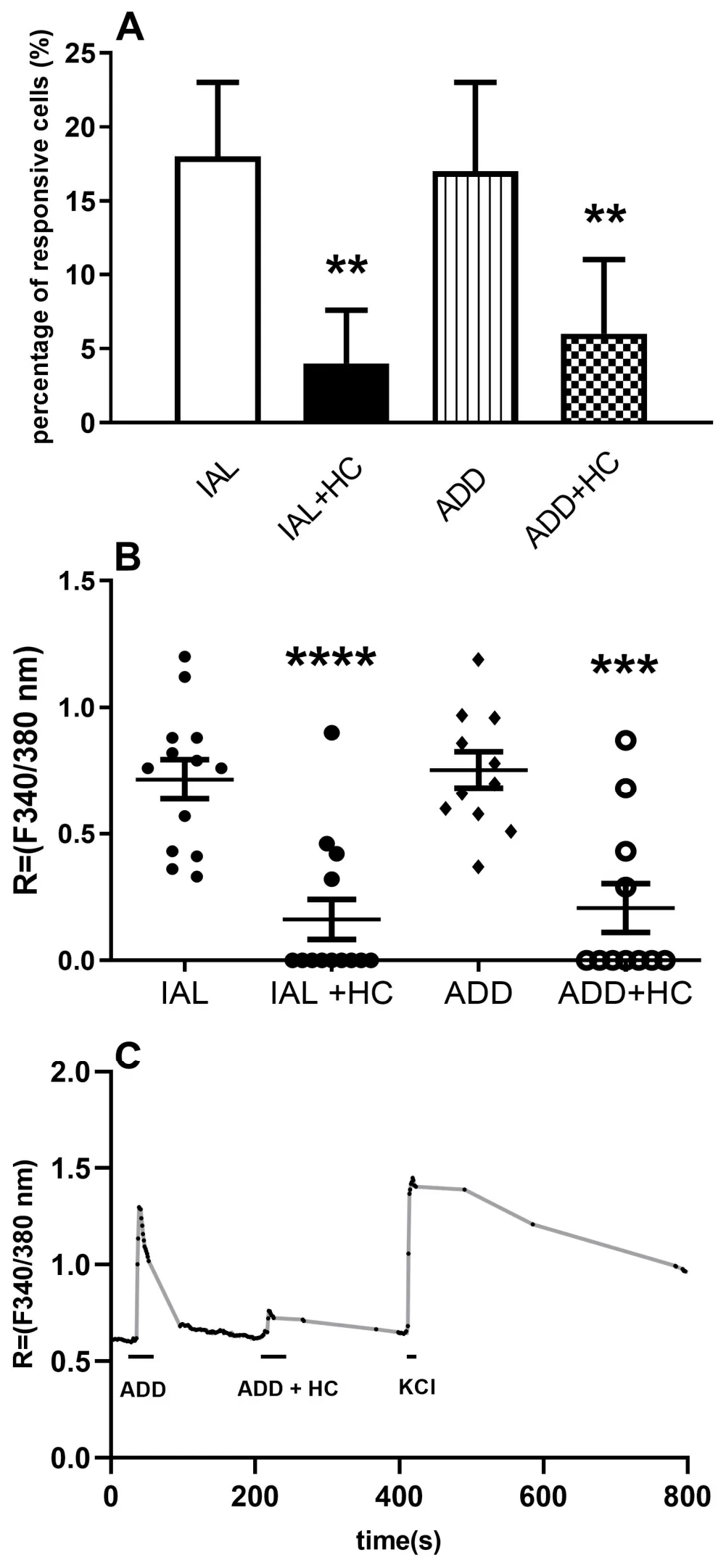
Effect of isoalantolactone (IAL) and acetoxydihydrodamsin (ADD) in cultured TG neurons. Increase in R = 340/380 fluorescence in Fura-2-loaded cultured trigeminal neurons. (**A**): Percentage of responsive cells to IAL, IAL + HC-030031, ADD, and ADD + HC-030031 is presented. Each column represents mean ± SEM of N = 65–100 cells per group. ** *p* < 0.01, one-way ANOVA with Tukey’s post hoc test. (**B**): Change in fluorescence ratio (R = F_340_/F_380_) is presented after compound administration. *** *p* < 0.001, **** *p* < 0.0001. (**C**): Original recording from ADD-sensitive neurons, effect of HC-030031 on ADD-induced Ca^2+^ transients and effect of KCl on same neuron. Data were obtained from three independent primary trigeminal ganglion cultures, each prepared from 3 neonatal mice.

In the first series of experiments, the percentage of neurons responding with Ca^2+^ influx to 40 μM isoalantolactone and acetoxydihydrodamsin was determined. The response to both compounds developed after a 15–30 s latency and lasted for 30–50 s. The percentages of isoalantolactone- and acetoxydihydrodamsin-responsive cells were 18 ± 5% (13 out of 72) and 17 ± 6% (11 out of 65), respectively. The respective R values (F_340_/F_380_) corresponding to the extent of the response were 0.71 ± 0.07 and 0.75 ± 0.07. The co-administration of the selective TRPA1 antagonist HC-030031 (10 μM) with isoalantolactone and acetoxydihydrodamsin significantly decreased both the percentage of responsive cells (4 ± 3.6%, 4 out of 100, and 6 ± 5%, 4 out of 66) and the response magnitudes (R values: 0.16 ± 0.07 and 0.20 ± 0.09, respectively) (Figure 3).

### 2.3. Binding Mode of Ligands

There is no experimentally determined binding mode for sesquiterpene-bound TRPA1; therefore, re-docking validation can only be performed with known covalent agonists, such as JT010 and benzyl isothicyanate (BITC), for which experimental structures are available. This re-docking validation was performed in our previous study [40], achieving experimental-like structures. For the present study, the same docking protocol was used as in [40], and the first-ranked binding modes are reported and discussed.

Neoambrosin, a structurally similar compound to damsin, was previously reported as a partial agonist in vitro [41] and docked to the electrophilic agonist binding site of TRPA1 [41]. The same receptor binding site was used for the focused docking of acetoxydihydrodamsin and other sesquiterpene lactone compounds in the present study. AITC was used as a strong reference against [42].

**Figure 4:**
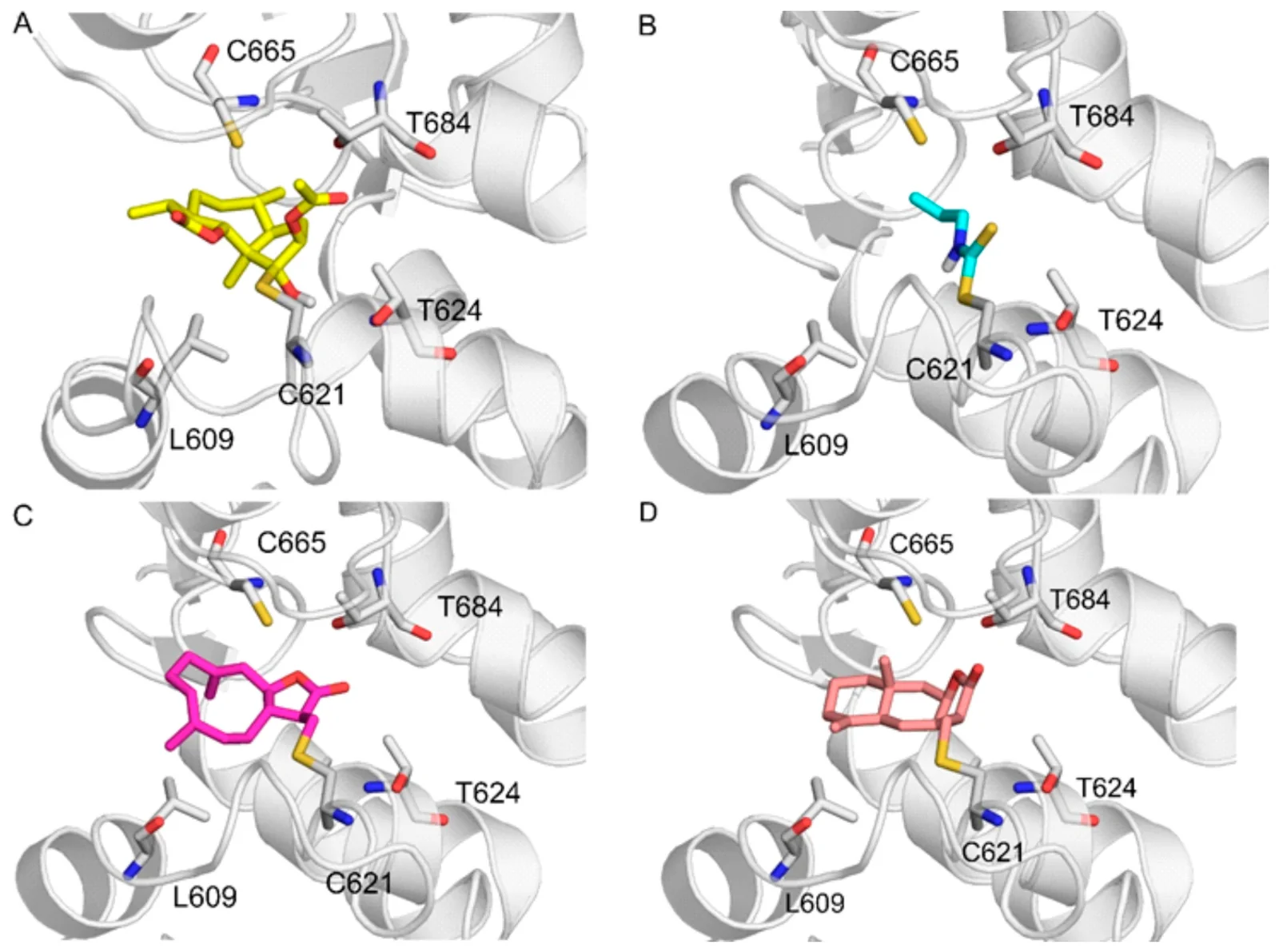
The binding of acetoxydihydrodamsin (**A**), AITC (**B**), costunolide (**C**), and isoalantolactone (**D**) to the TRPA1 receptor. The ligands are shown in yellow, teal, purple, and pink as all-atom representation sticks, respectively. The TRPA1 receptor is shown as a gray cartoon, and the interacting amino acids are shown as gray all-atom representation sticks and labeled.

Our molecular docking studies revealed that all four agonists can bind covalently to C621 and interact with the C665 side chain, a feature commonly observed for electrophilic TRPA1 agonists [40,43,44]. In addition to the covalent bond, acetoxydihydrodamsin forms polar interactions with T624 and T684 and a hydrophobic interaction with L609 (Figure 4). 

This interaction pattern is similar to that of neoambrosin [41], which has a similar scaffold. Acetoxydihydrodamsin has a ketone covalent warhead; costunolide and isoalantolactone are Michael acceptors. The binding modes of the three compounds are similar to those of the strong agonist AITC, resulting in a considerably favorable binding free energy (Table 1). 

## 3. Discussion

In the present study, we demonstrate that sesquiterpene lactones act as agonists of the TRPA1 ion channel, but not of TRPV1, in vitro in receptor-expressing CHO cells and sensory neurons. We provide the first data for the activating effect of acetoxydihydrodamsin on TRPA1 ion channel function in these experimental systems. 

Acetoxydihydrodamsin increased ^45^Ca^2+^ uptake in TRPA1-expressing CHO cells in a concentration-dependent manner and elevated intracellular calcium levels in native trigeminal neurons. The selective TRPA1 antagonist HC-030031 significantly inhibited the effect of acetoxydihydrodamsin in both receptor-expressing CHO cells and sensory neurons. The results from fluorescent calcium imaging measurements, including the percentage of responding cells and the time course of calcium transients, are consistent with our previous findings using the reference TRPA1 agonist AITC [45,46,47].

The mechanism of the sesquiterpene activation of TRPA1 was investigated using molecular docking. Sesquiterpenes were able to bind covalently to the conserved nucleophilic C621 residue in the intracellular domain of the protein [40,43,44]. Costunolide, isoalantolactone, and acetoxydihydrodamsin act as covalent, selective agonists of TRPA1 but do not activate TRPV1. The electrophilic α-methylene-γ-lactone warhead of costunolide and isoalantolactone renders them strong Michael acceptors, a common feature of electrophilic natural products that are reactive with thiols [30]. Although acetoxydihydrodamsin lacks the α-methylene-γ-lactone moiety, it is still capable of covalent interaction with the C621 residue, most likely via a non-Michael addition involving its ketone functional group [48]. This observation indicates that the presence of the α-methylene-γ-lactone moiety is not an exclusive structural requirement for TRPA1 activation by natural products. Our conclusion that acetoxydihydrodamsin can bind covalently to TRPA1 is inferred from computational docking and pharmacological inhibition in the present study and is supported by the thiol reactivity of ketone warheads [49,50,51,52], the ability of electrophilic agonists to bind covalently to the C621 residue of TRPA1 [44,53,54] and the docking studies of the sesquiterpene compounds neoambrosin [35] and cinnamodial [55] binding to C621 of TRPA1. A limitation of the present study is that psilostachyin and peruvin were tested only at the screening concentration of 40 µM. Therefore, the absence of significant TRPA1 or TRPV1 activation under these conditions should not be interpreted as evidence of complete inactivity, since agonistic effects at higher concentrations cannot be excluded. Full concentration–response studies would be required to determine whether these compounds are inactive or merely have lower potency at these channels.

The 15–30 s latency of receptor activation observed for the investigated sesquiterpene lactones may be explained by the intracellular localization of the binding site, as the compound needs to enter the cell and access the intracellular A-loop before binding can occur [40]. The relatively prolonged (30–50 s) activation might support a covalent binding mechanism, which is typically associated with a slow off-rate, whereas receptor desensitization may result from substantial Ca^2+^ influx. After KCl administration, the cells were reactivated, indicating preserved cellular viability.

The covalent modification of TRPA1 has previously been shown to induce initial activation followed by desensitization [31,32], which may contribute to the allergic activity of ragweed. However, this mechanism may also underlie potential therapeutic effects through the desensitization of nociceptive pathways. This is particularly relevant given that ragweed has traditionally been regarded as a plant without medicinal value. 

Sesquiterpene lactones have been reported in Asteraceae pollen [56]. Ragweed pollen concentration correlates with the severity of seasonal rhinoconjunctivitis and bronchial symptoms in sensitized patients [57]. In addition, these compounds have long been implicated in airborne contact dermatitis, and sesquiterpene lactone-sensitive individuals frequently react to *A. artemisiifolia* extracts [58]. Therefore, if electrophilic sesquiterpene lactones are present in ragweed pollen and become available at the airway surface, they may contribute to acute exposure-related effects not only through immune mechanisms but also via the activation of TRPA1-expressing sensory afferents [59]. Exposure to ragweed can cause allergic rhinitis [60], a disease characterized by type 2 inflammation mediated by immunoglobulin E [61]. The immune cell-rich submucosa of the nasal cavity responds to allergen exposure via the cross-linking of mast cell and basophil surface-bound immunoglobulin E; the mast cells, in turn, degranulate and release histamine and leukotrienes. Additionally, the nerve fibers in the nasal cavity can release SP and CGRP, [62], and both neurotransmitters can regulate T helper 17 and regulatory T cell differentiation, which mediate pathogenesis in allergic rhinitis [63]. The SP- and CGRP-mediated chemoattraction of immune cells causes vasodilation, plasma extravasation, glandular secretion, and an enhancement in allergic reaction [62]. SP can also contribute to pathogenesis by increasing the production of regulatory cytokines, including interleukins 1, 3, 5, and 6; tumor necrosis factor α; and interferon γ [62]. CGRP can cooperate with alarmins and interleukins 25 and 33, in turn recruiting type 2 innate lymphoid cells (ILC2) and localized eosinophil accumulation [64].

Several structurally related sesquiterpene lactones have demonstrated anti-inflammatory activities in preclinical models affecting the skin, joints, and mucosal tissues. Costunolide and isoalantolactone attenuate experimental osteoarthritis, improve colitis models, and reduce inflammatory responses in airway and gastric mucosal models [65,66,67]. Our results and the above-mentioned studies could possible imply that ragweed sesquiterpene lactones may represent compounds with dual relevance: contributing to acute irritation while also possessing pharmacological potential. The TRP affinity of ragweed compounds may shed new light on the perception of this species, which is generally regarded as an allergenic plant with no medicinal value, and could also pivot research on the plant in a new direction.

## 4. Materials and Methods

### 4.1. Compound Isolation

Acetoxydihydrodamsin, peruvin, and psilostachyin (Figure 1) were isolated from the aerial parts of *A. artemisiifolia* [37]. Identification was performed by means of NMR spectroscopy (Supplementary Material Figures S1–S3). The identity and chemical homogeneity of the isolated compounds were confirmed by ^1^H and phase-edited ^13^C NMR spectroscopy. No substantial unassigned resonances, other than residual solvent signals, were observed in the ^1^H NMR spectra; however, quantitative purity values were not determined. Costunolide was obtained from Merck KGaA (Darmstadt, Germany), and isoalantolactone was purchased from MedChemExpress LLC (Monmouth Junction, NJ, USA). Based on the manufacturers’ certificates of analysis, purity was ≥96%. Capsaicin and AITC (Sigma, St. Louis, MO, USA) were dissolved in DMSO to obtain a 10 mM stock solution. Further dilutions were prepared in ECS to reach the final concentrations. DMEM, horse serum, fetal bovine albumin, and newborn calf serum were purchased from Gibco (Grand Island, NY, USA). Collagenase, deoxyribonuclease I, poly-D-lysine, and NGF were purchased from Sigma (St. Louis, MO, USA).

### 4.2. Radioactive Calcium-45 Uptake Experiments in CHO Cells Expressing Cloned Rat TRPV1 and TRPA1 Receptors 

A human TRPA1 cDNA clone was obtained from the Life Technologies CloneRanger collection (clone ID: 100016279). A human TRPV1 cDNA clone was obtained from the OriGene Inc. (TrueClone ID: TC109883) (Rockville, MD, USA). cDNA was inserted between the CMV promoter and the bovine growth hormone polyA region in pT3CMV vector (unpublished). pT3CMV is a derivate of the pT2/BH Sleeping Beauty transposon vector (Addgene, ID: 26556). CHO cells were co-transfected with vectors containing human TRPA1 and TRPV1 cDNA and the pCMVSB100x vector expressing the transposase. The presence of the functional human TRP receptors was checked by Fura-2 microfluorimetry and Fluo-4 flowcytometry [68]. CHO cells stably expressing TRPA1 or TRPV1 were plated onto Microwell Minitrays in 15 µL culture medium as described previously [47,69]. On the following day, the cells were washed five times with calcium-free Hank’s solution (pH 7.4). Cells were then incubated for 2 min at room temperature in 10 µL buffer containing the respective test compound or positive control agonist together with ^45^Ca^2+^ at a concentration of 200 µCi/mL (specific activity: 1.3 Ci/mmol; Amersham, UK). The 2 min incubation period was selected according to our previously established radioactive calcium uptake protocol for TRP receptor-expressing cells [47,69], allowing for the rapid measurement of receptor-mediated Ca^2+^ influx while limiting secondary effects associated with prolonged stimulation.

AITC (200 µM) and capsaicin (330 nM) served as positive controls for TRPA1 and TRPV1 activation, respectively. Basal isotope retention was determined in cells exposed to isotope-containing buffer without agonist. Test compounds were costunolide (40 µM), isoalantolactone (40 µM), psilostachyin (40 µM), peruvin (40 µM) or acetoxydihydrodamsin (1, 10, 40, 200 µM). Following stimulation, cells were washed five times with extracellular solution (ECS) containing (in mM) NaCl 160, KCl 2.5, CaCl_2_ 1, MgCl_2_ 2, HEPES 10, and glucose 10 at pH 7.3. The residual liquid was evaporated, and the retained isotope was collected in 15 µL of 0.1% SDS. Radioactivity was determined in 2 mL scintillation fluid using a Packard Tri-Carb 2800 TR scintillation counter. The data obtained in the CHO cell experiments are presented as the mean ± SEM from three independent experiments, each performed in triplicate.

### 4.3. Primary Cultures of Trigeminal Ganglion (TRG) Neurons 

Cell cultures were prepared from the trigeminal ganglia of 1-4-day-old NMRI mouse pups. The ganglia were cut and placed into ice-cold phosphate-buffered saline (PBS) and incubated for 35 minutes at 37 °C in PBS containing collagenase (Type XI, 1 mg/mL), then deoxyribonuclease I (1000 units/mL) for 8 min. After PBS washing, they were dissociated by trituration. Individual neurons were plated on poly-D-lysine-coated glass coverslips and grown in a nutrient-supplemented medium (180 mL Dulbecco′s Modified Eagle Medium (D-MEM), 20 mL horse serum, 20 mL bovine albumin, 2 mL insulin–transferrin–selenium-S, 3.2 mL putrescin dihydrochloride (100 μg/mL), 20 μL triiodo-thyronine (0.2 mg/mL), 1.24 mL progesterone (0.5 mg/mL), 100 μL penicillin, 100 μL streptomycin). Neurons were maintained at 37 °C in a humidified atmosphere with 5% CO_2_, and nerve growth factor (NGF, 200 ng/mL) was added as described earlier [47]. Calcium imaging experiments were performed using three independent primary trigeminal ganglion cultures. Each culture was prepared from the trigeminal ganglia of at least 3 neonatal NMRI mice.

### 4.4. Ratiometric Technique of Measurement of Intracellular Free Calcium Concentration ([Ca^2+^]_i_) with Fluorescent Indicator Fura-2 AM Dye

One- to three-day-old neuronal cultures were stained with 1 μM of fluorescent Ca^2+^ indicator dye, fura-2-AM (Molecular Probes) in an incubation solution (containing (in mM): NaCl, 122; KCl, 3.3; CaCl_2_, 1.3; MgSO_4_, 0.4; KH_2_PO_4_, 1.2; HEPES, 25; glucose, 10; (pH 7.3)) for 60 min at 37 °C. After 5 min of washing in extracellular solution (ECS) containing (in mM) NaCl, 160; KCl, 2.5; CaCl_2_, 1; MgCl_2_, 2; HEPES, 10; and glucose, 10 (pH 7.3), calcium transients of neurons to chemical compounds were examined with microfluorimetry as described elsewhere [47]. The rapid change in solutions was controlled by a fast-step perfusion system (VC-77SP, Warner Instrument Corporation, Harvard Apparatus GmbH, Holliston, MA, USA). An Olympus LUMPLAN FI/x20 0.5 W water immersion objective and a digital camera (CCD, SensiCam PCO, Germany) connected to a computer were used to acquire fluorescence images of up to 12 dye-loaded cells per plate. Cells were illuminated alternately with 340 and 380 nm light (for 50 to 200 ms each) generated by a monochromator (Polychrome II., Till Photonics, Grafelfing-Lochham, Germany) under the control of Axon Imaging Workbench 2.1 (AIW, Axon Instruments, San Jose, CA, USA). We measured the emitted light > 510 nm. The ratio value, R = F_340_/F_380_, was monitored continuously (rate: 1 Hz). The R value is the ratio of fluorescence intensities measured at two different excitation or emission wavelengths, which reflects changes in intracellular calcium concentration. The R values were generated by AIW 2.1 software, then processed by GraphPad Prism software version 8.0.1. Calcium transients were investigated after the administration of isoalantolactone (40 µM) and acetoxydihydrodamsin (40 µM), as well as their combination with HC-030031. The fluorescence ratio (F340/F380) was monitored for at least 15 s at the beginning of each measurement to establish the baseline R value for each neuron individually. The response amplitude was calculated as the difference between the maximum R value reached following stimulation and the corresponding baseline R value. A neuron was considered responsive when stimulation induced an increase in the F340/F380 ratio of at least 0.1 R.

### 4.5. Statistical Analysis

The data reported in the in vitro investigation are the means ± SEM of at least three independent experiments. Statistical analysis was performed by a one-way ANOVA with Tukey’s post hoc test or Dunnett’s post hoc test; in all cases, *p* < 0.05 was considered statistically significant.

### 4.6. Molecular Docking Studies

Ligand preparation. Allyl isothiocyanate (AITC, used as a reference ligand), costunolide, isoalantolactone, and acetoxydihydrodamsin were built in the 2D sketcher of FITTED Forecaster Build: 6383 ([70,71], https://molecularforecaster.com/academic-license/, access on 16 August 2026) saved as an sdf file and converted to a mol2 file with the Convert function. Rotatable bonds were set up in Smarts in the graphical user interface of FITTED.

Target preparation. A previously prepared human TRPA1 receptor [40] was used as the target. Chain A of the Protein Data Bank (PDB [72]) structure PDB:6pqp [73] was kept for the homotetramer structure to decrease computational cost; missing residues were corrected by homology modeling with SWISS-MODEL ([53], https://swissmodel.expasy.org/, access on 16 August 2026). The resulting structure was energy-minimized using GROMACS 2020.4 ([40,72], https://manual.gromacs.org/current/download.html, access on 16 August 2026). The default settings for the FITTED built-in target preparatory steps were used. The Prepare algorithm converts the protein into a mol2 file, adds hydrogen atoms, and optimizes amino acid side chain conformations. The original ligand of PDB:6pqp was used to identify the binding site in the Process algorithm. 

Docking simulation. The covalent docking mode of FITTED was used with default settings. The covalent warhead was recognized for all four ligands. C621 was specified as the covalently binding amino acid, as a similar ligand to damsin was previously docked to the same binding site by another group [41]. Five docking runs were carried out in each case. The results were ranked by their calculated binding energy, and only the most favorable rank was printed as an SDF file.

## Figures and Tables

**Table 1:** Comparison of raw scoring function output by FITTED and warhead type along tested compounds and one reference compound.

	Acetoxydihydrodamsin	AITC	Costunolide	Isoalantolactone
ΔG_binding,FD_ (kcal/mol)	−45.0	−77.7	−47.3	−51.5
Warhead type	Ketone	Isothiocyanate	Michael acceptor	Michael acceptor

## Data Availability

The original contributions presented in this study are included in the article/Supplementary Material. Further inquiries can be directed to the corresponding author.

## Supplementary Materials

The following supporting information can be downloaded at https://www.mdpi.com/article/10.3390/ph19091404/s1, Figure S1: ^1^H and ^13^C NMR spectra of acetoxydihydrodamsin; Figure S2: ^1^H and ^13^C NMR spectra of peruvin; Figure S3: ^1^H and ^13^C NMR spectra of psilostachyin.

## Abbreviations

The following abbreviations are used in this manuscript:

AITC	allyl isothiocyanate
CHO cells	Chinese Hamster ovary cells
D-MEM	Dulbecco’s modified Eagle medium
ECS	extracellular solution
FITTED	flexibility induced through targeted evolutionary description
GROMACS	GROningen MAchine for Chemical Simulations
HEPES	4-(2-hydroxyethyl)-1-piperazineethanesulfonic acid
NMR	nuclear magnetic resonance spectroscopy
NMRI	Naval Medical Research Institute
PBS	phosphate-buffered saline
TRPA1	transient receptor potential ankyrin 1
TRPV1	transient receptor potential vanilloid 1
