# Supplementary material for: Sesquiterpene Lactone Derivatives of Ambrosia artemisiifolia Act as Agonists of the Transient Receptor Potential Ankyrin 1 Ion Channel
Source: Pharmaceuticals (Basel). 2026 Sep 6;19(9):1404. doi: 10.3390/ph19091404 (PMC13610691; doi:10.3390/ph19091404)
Supplement: Supplementary file 1 [file pharmaceuticals-19-01404-s001.zip › pharmaceuticals-4538423-supplementary.pdf]

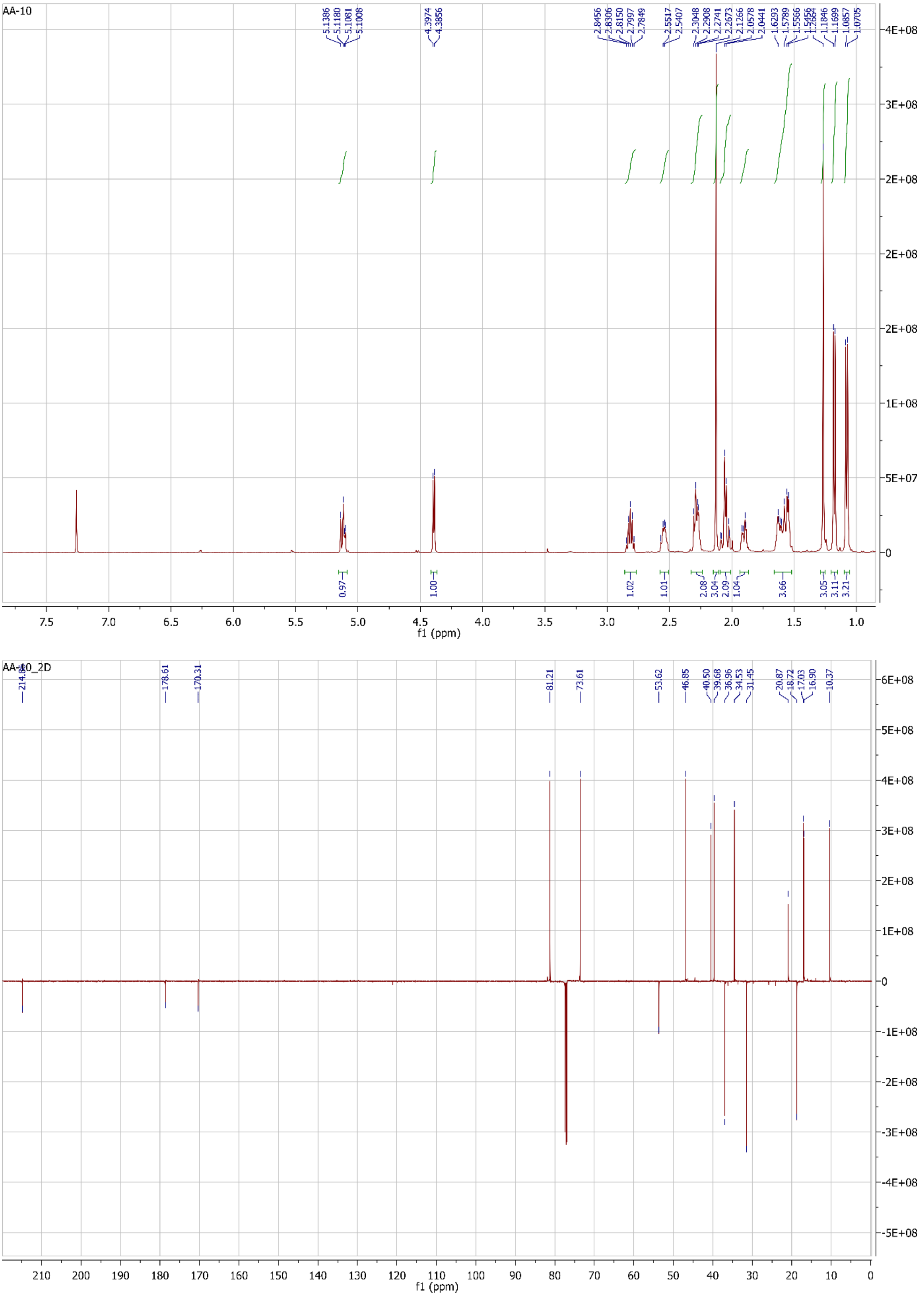

Figure S1. ^{1}H and ^{13}C NMR spectra of acetoxydihydrodamsin

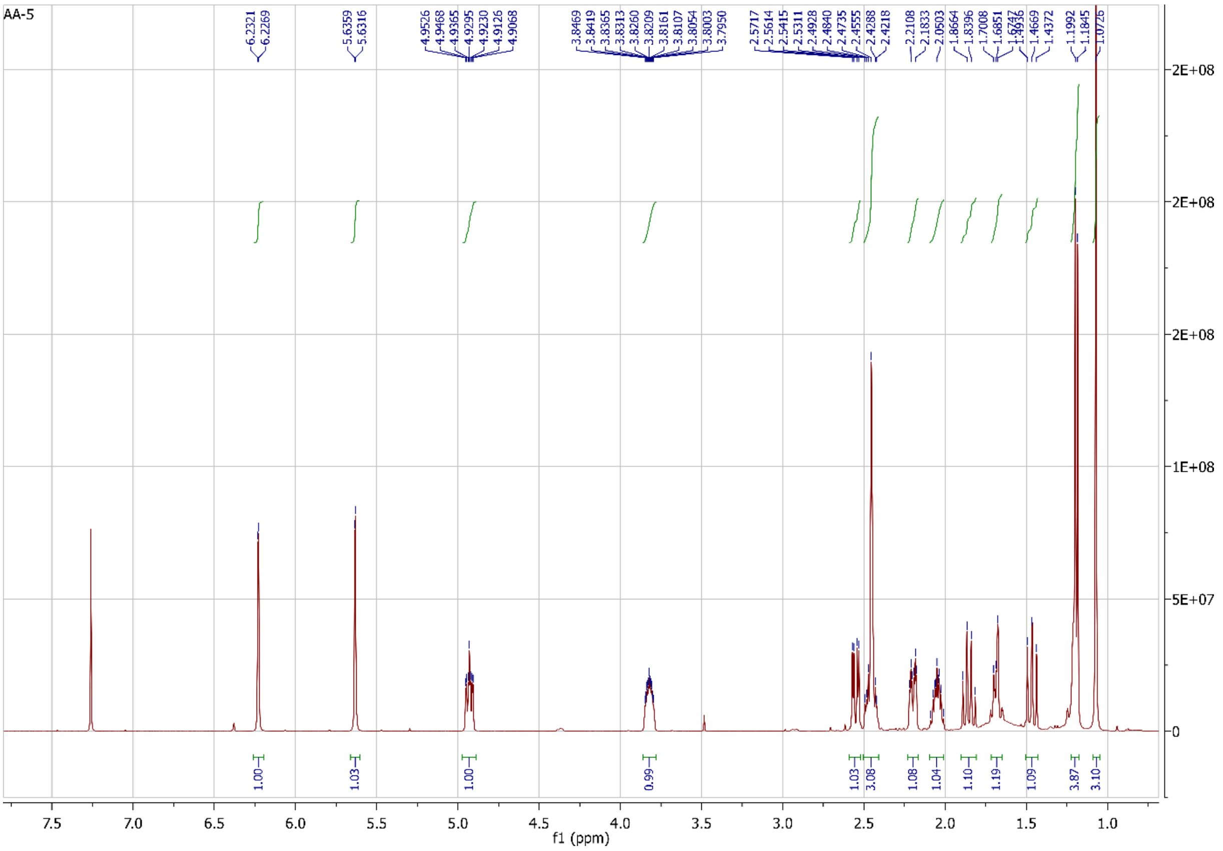

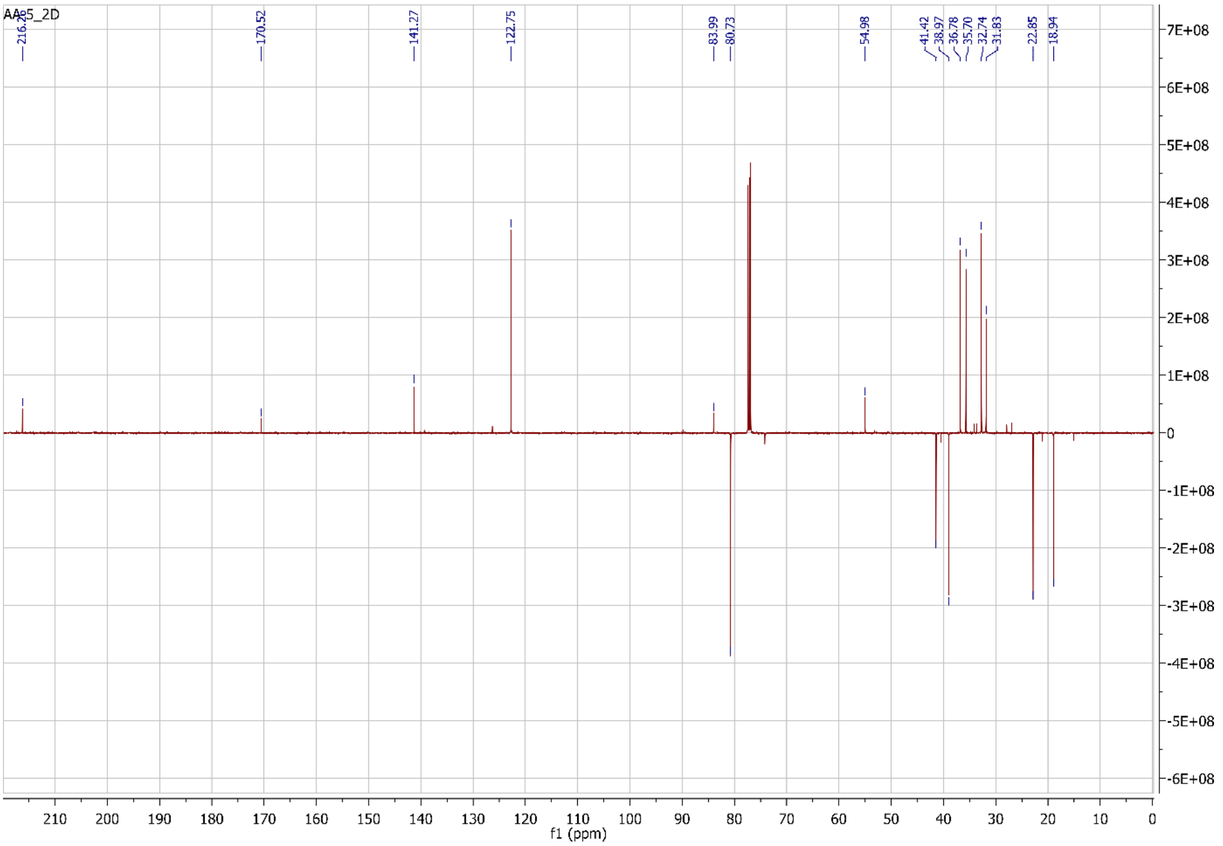

Figure S2. ^{1}H and ^{13}C NMR spectra of peruvin

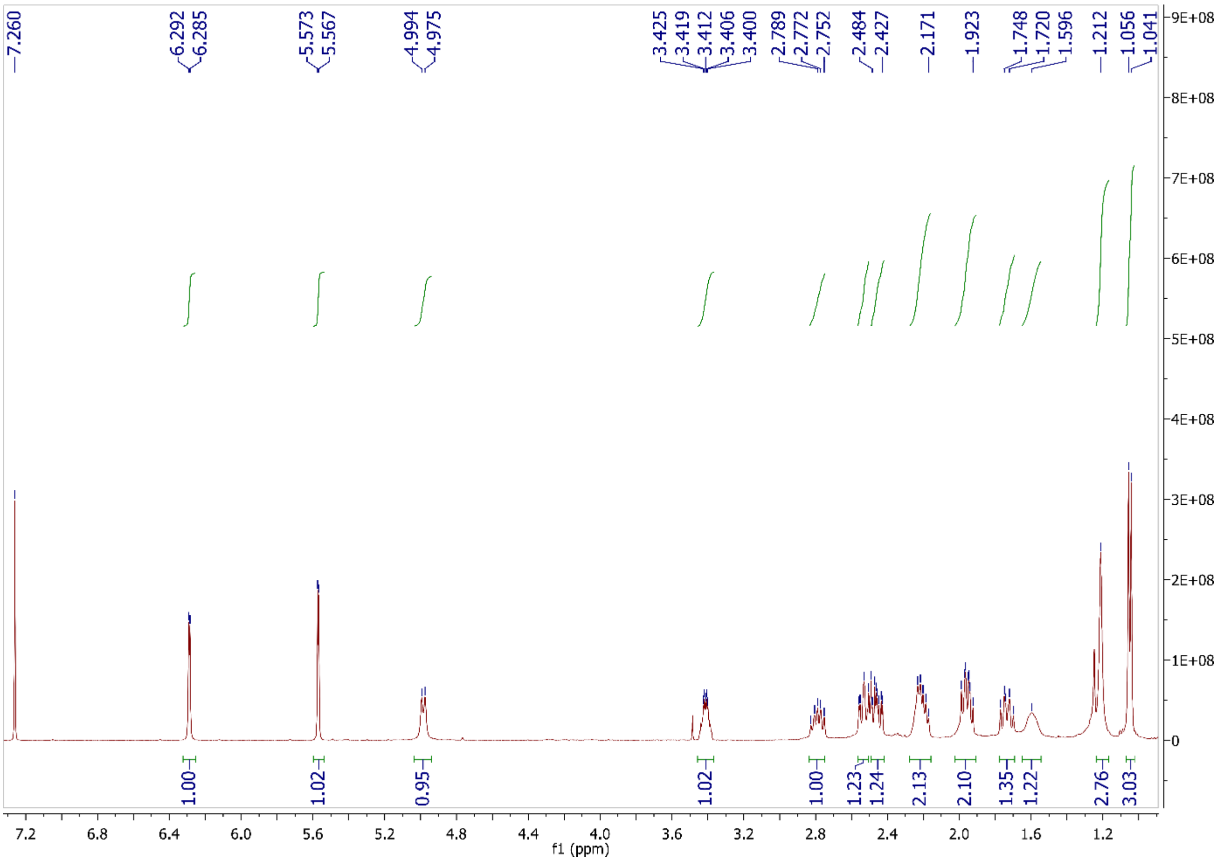

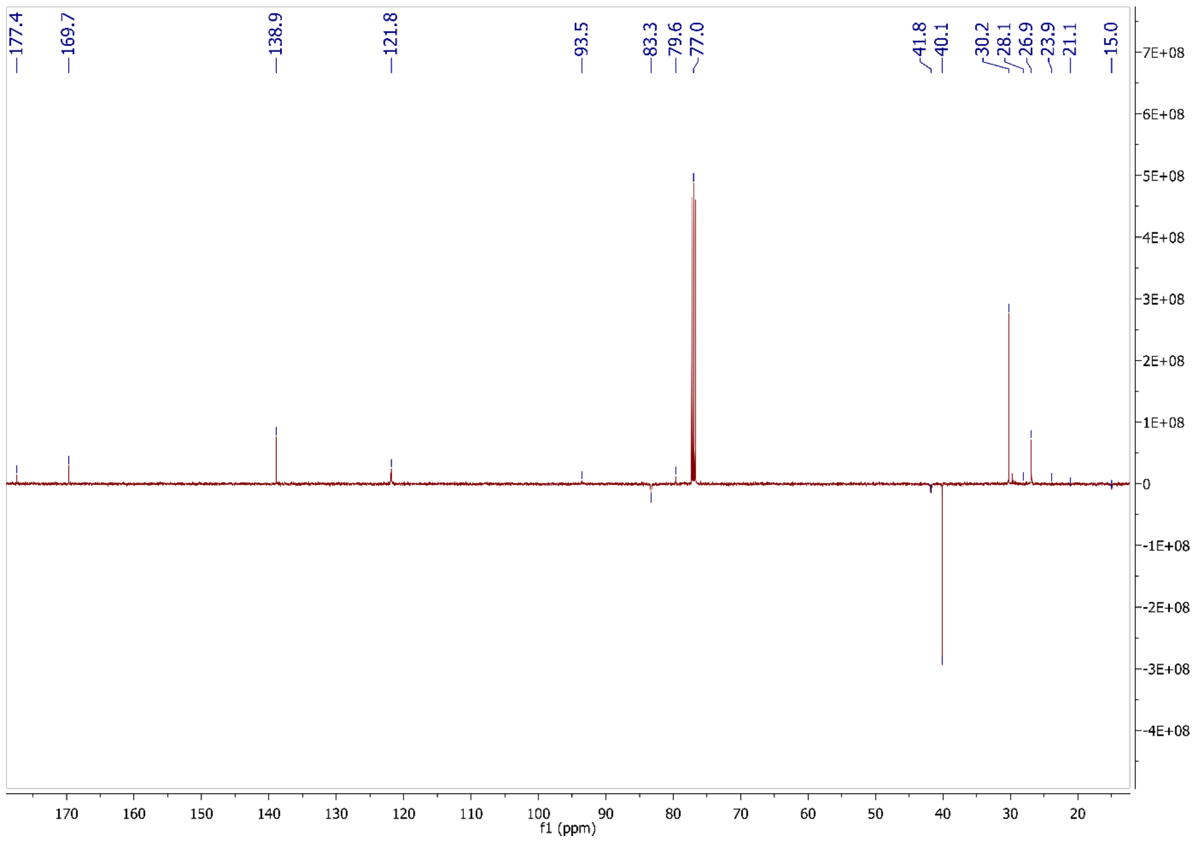

Figure S3. ^{1}H and ^{13}C NMR spectra of psilostachyin
